# Potential Therapeutic Effect of Natural Killer Cells on Doxorubicin-Resistant Breast Cancer Cells *In Vitro*

**DOI:** 10.1371/journal.pone.0136209

**Published:** 2015-08-21

**Authors:** Mi-Hye Hwang, Xiu Juan Li, Jung Eun Kim, Shin Young Jeong, Sang-Woo Lee, Jaetae Lee, Byeong-Cheol Ahn

**Affiliations:** Department of Nuclear Medicine, Kyungpook National University School of Medicine, Daegu, Republic of Korea; AntiCancer Inc., UNITED STATES

## Abstract

**Objective:**

The aim of this study was to explore the therapeutic effect of natural killer (NK) cells on human doxorubicin-sensitive and resistant breast adenocarcinoma.

**Methods:**

Human doxorubicin-sensitive and resistant breast cancer cell lines (MCF-7 and MCF-7/ADR) were tagged with renilla luciferase (Rluc) (MCF-7/RC and MCF-7/ADR/RC). NK cells were tagged with enhanced firefly luciferase (effluc) using a recombinant retrovirus transfection (NKF). Expression of Rluc, effluc, and NK cell surface markers CD16, CD56 as well as death receptors, DR4 and DR5, were assessed by using flow cytometry. *In vitro* cytotoxic effect of NK to MCF-7 and MCF-7/ADR was measured and *in vivo* bioluminescence imaging was also performed to visualize MCF-7/RC, MCF-7/ADR, and NKF in an animal model.

**Results:**

NK92-MI, MCF-7, and MCF-7/ADR cells were successfully labeled with Rluc or effluc. Both the target breast cancer cells (with Rluc) and therapeutic NK cells (with effluc) were noninvasively visualized in nude mice. Doxorubicin-resistant breast cancer cells (MCF-7/ADR) presented a higher expression of DR5 and were more sensitive to NK cells compared with doxorubicin-sensitive breast cancer cells (MCF-7).

**Conclusion:**

The results of present study suggest that NK cell therapy has a therapeutic effect on doxorubicin-sensitive and resistant breast cancer cells.

## Introduction

Chemotherapy resistance is one of the challenges in local and metastatic breast cancer [1]. Doxorubicin (DOX) has been clinically approved as a chemotherapeutic agent, owing to its wide anticancer spectrum and superior cytotoxicity [2]. Unfortunately, cancer cells, including breast cancer cells, have been reported to express multi-drug resistance genes, including the gene encoding for P-glycoprotein after DOX administration [3]. Response rates to single DOX treatment range from 43% in previously untreated patients to 28% in patients previously exposed to the drug, indicating that DOX exposure induces a growing resistance to the drug [4]. Therefore, application of other systematic therapeutic strategies is critical to overcome drug-resistance in breast cancer.

Recent data suggest that natural killer (NK) cells, which are a type of cytotoxic lymphocyte such as T and B cells and a key component of the innate immune system, is capable of mediating cytotoxicity against tumor cells, including breast cancer [5–7]. Herberman RB summarized the important role of NK cells against tumors as well as other fields. [8].RK Pachynski et al reported NK cells recruited by chemoattractment chemerin inhibited melanoma growth [9].There are two major mechanisms of cytotoxicity of NK cells to induce cell death which are perforin/multiple granzymes-dependent necrosis and apoptosis through at least three death ligands (TNF-α, FasL, and TRAIL), each of which interacts with specific receptors on the target cell surface [10–12]. It was reported that the surface of NK cells was functionalized with TRAIL liposomes to kill cancer cells in *in vitro* models of lymph node micrometastasis through binding death receptors DR4 and DR5 [13]. MJ Mitchell et al were also inspired by the cytotoxic activity of NK cells to use circulating leukocytes presented the TRAIL to target and kill colon and prostate cancer cells in the blood [14]. Although many studies have explored its efficacy in anticancer therapy, the effect of NK cells in human drug-resistant breast cancer remains unclear.

In this study, a powerful molecular imaging technique, using bioluminescent reporter genes, which allow the non-invasive detection of biological processes at the cellular and subcellular levels in intact living subject [15], was used to monitor the effect of NK cells on DOX-resistant breast cancer cells.

## Materials and Methods

### Cell lines

Human breast cancer cell line, MCF-7, and the DOX-resistant cell line, MCF-7/ADR, were kindly provided by J.A Kim (YeungNam University, Gyeongsan, Republic of Korea) as used previously [16].

MCF-7/ADR cells were grown in Dulbecco’s Modified Eagle Medium (DMEM)-high glucose (Hyclone, Logan, UT, USA) containing 10% fetal bovine serum (FBS, Hyclone) and 1% penicillin-streptomycin at 37°C in a 5% CO_2_ atmosphere. MCF-7 and MCF-7/ADR were transfected with a recombinant lentivirus with a plasmid containing both renilla luciferase (Rluc) and mCherry driven by a cytomegalovirus (CMV) promoter (Lenti-CMV-Rluc-mCherry). Cells expressing Rluc and mCherry were sorted by using flow cytometry (FACSorter; BD Biosciences, San Jose, CA, USA). The established stable cell lines expressing both Rluc and mCherry genes are herein referred to as MCF-7/RC and MCF-7/ADR/RC cells. Mcherry expression was checked under microscopy in MCF-7/RC and MCF-7/ADR/RC cells.

The human NK cell line (NK92-MI) was obtained from the American Type Culture Collection (ATCC, Rockville, MD, USA). NK92-MI cells were incubated in alpha modification of Eagle’s minimum essential medium (α-MEM; GIBCO, Carlsbad, CA, USA) supplemented with 2 mM L-glutamine, 0.2 mM inositol, 0.02 mM folic acid, 0.01 mM 2-mercaptoethanol, 12.5% FBS (Hyclone), 12.5% horse serum (GIBCO), and 1% penicillin-streptomycin at 37°C in a 5% CO_2_ atmosphere. The cells were transfected with a recombinant retrovirus with a plasmid containing both enhanced firefly luciferase (effluc) and thy1.1 driven by a long terminal repeat (LTR) promoter (Retro-LTR-effluc-thy1.1). NK cells expressing effluc and thy1.1 were sorted by magnetic cell sorting (MACS; Miltenyi Biotech, Auburn, CA, USA) for thy1.1 positive cells. For magnetic cell sorting, cells were re-suspended in 0.1% bovine serum albumin (BSA)-phosphate buffered saline (PBS) and labeled with the CD90.1 antibody (Miltenyi Biotech). The established stable cell lines expressing both effluc and thy1.1 gene are herein referred to as NKF cells.

### Immunofluorescent staining

For confocal microscopic analysis, NK92-MI and NKF cells were seeded at 2 × 10^4^ cells per chamber on Laboratory-Tek German borosilicate cover glass with eight chambers (Nunc, Rochester, NY, USA) and incubated for 24 h in growth medium. The cells were washed twice with 200 μL PBS. Subsequently, the cells were fixed and permeabilized with Fixation/Permeabilization buffer (BD Bioscience, San Jose, CA, USA) for 30 min at 4°C. The cells were then washed with 200 μL 1× BD wash buffer and incubated with phycoerythrin (PE)-conjugated anti-mouse CD90.1 (BD Bioscience) antibody at room temperature for 1 h followed by three washes with 200 μL 1× BD wash buffer. The slides were mounted with Vectashield Mounting Medium (Vector Laboratories, Burlingame, CA, USA) and covered with glass cover slips. Confocal scanning laser microscopy was performed using a Zeiss LSM 510 instrument (Carl Zeiss, Oberkochen, Germany) with a 40× oil objective, as indicated. The images were processed using Aim Image Examiner software (Carl Zeiss).

### Flow cytometry Analysis

NK cell lines (NK92-MI and NKF) and breast cancer cell lines (MCF-7 and MCF-7/ADR) were analyzed by flow cytometry (FACScalibur, BD Biosciences) using the following antibodies: FITC-labeled anti-human CD56 (BD Biosciences), PE-labeled anti-human CD16 (BD Biosciences), PE-labeled anti-DR4 (eBioscience, San Diego, CA, USA), and PE-labeled anti-DR5 (eBioscience). Unspecific antibody binding was analyzed by staining with isotype-match FITC- and PE-labeled control antibodies (BD Biosciences).

### Cytotoxicity Assay

To evaluate the NKF-induced apoptosis in MCF-7/RC and MCF-7/ADR/RC cells, effector and target cells were co-cultured in growth medium at different effector to target ratios (2:1, 5:1, and 10:1) in white and clear bottom 96-well plate and bioluminescence imaging (BLI) signals from Renilla luciferase reporter gene were measured at the indicated times using a microplate reader after adding coelenterazine which is the substrate of Renilla luciferase. (Molecular Devices, Sunnyvale, CA, USA). Cytotoxic activity of NK cells was also assessed using the CytoTox 96 Non-Radioactive Cytotoxicity Assay system (Promega, USA). 1x10^4^ MCF-7/RC and MCF-7/ADR/RC cells were cultured in a round-bottom, 96 well plate. The effectors NK cells were distributed in triplicate at effector:target (E:T) cell ratios from 1:1,2.5:1,5:1.After incubation at 37°C in 5% CO_2_ for 4hr, each supernatant was harvested and transferred into new paltes. Samples were measured using a microplate reader (Bio-Rad, Hercules, CA, USA). Data were expressed as at arbitrary fluorescent units. NK cell cytotoxicity was calculated using the following equation:

NK cell cytotoxicity(%)={(Experimental-Effector Spontaneous-Target Spontaneous)/(Target Maximum-Target Spontaneous)}X100%

### *In vivo* animal experiment

Specific pathogen-free six-week–old female BALB/c nude mice (Hamamatsu, Shizuoka, Japan) were used for *in vivo* studies. All animal experiments were conducted in accordance with the National Institutes of Health guidelines for the care and use of laboratory animals and approved by the Committee for Handling and Use of Animals of Kyungpook National University.

For evaluation of the functional expression of the Rluc gene *in vivo*, MCF-7/RC and MCF-7/ADR/RC cells in PBS were implanted subcutaneously into the left fore-flank (1 × 10^5^ cells), left hind-flank (3 × 10^5^ cells), and right hind-flank (9 × 10^5^ cells) of three mice. After tumor implantation, BLI was performed using the IVIS Lumina II imaging system (Caliper, Alameda, CA, USA), which included a highly sensitive CCD camera mounted on a light-tight specimen chamber. For evaluation of the functional expression of the effluc gene *in vivo*, 1 × 10^5^, 5 × 10^5^, and 2.5 × 10^6^ NKF cells were inoculated subcutaneously into the left fore-flank, left hind-flank, and right hind-flank of female Balbc/nude mice, respectively (n = 3). The bioluminescence images were took immediately after cells implantation. Ten minutes after intraperitoneal administration of D-luciferin (3 mg/mouse; Caliper), bioluminescence images were taken for 5 min using the IVIS Lumina II imaging system.

### Statistical Analysis

All numeric data are expressed as the mean ± standard deviation. Inter-group differences were assessed using a two-tailed Student’s *t*-test. P values <0.05 were considered statistically significant.

## Results

### Establishment of reporter gene expressing stable cell lines

**Fig 1:**
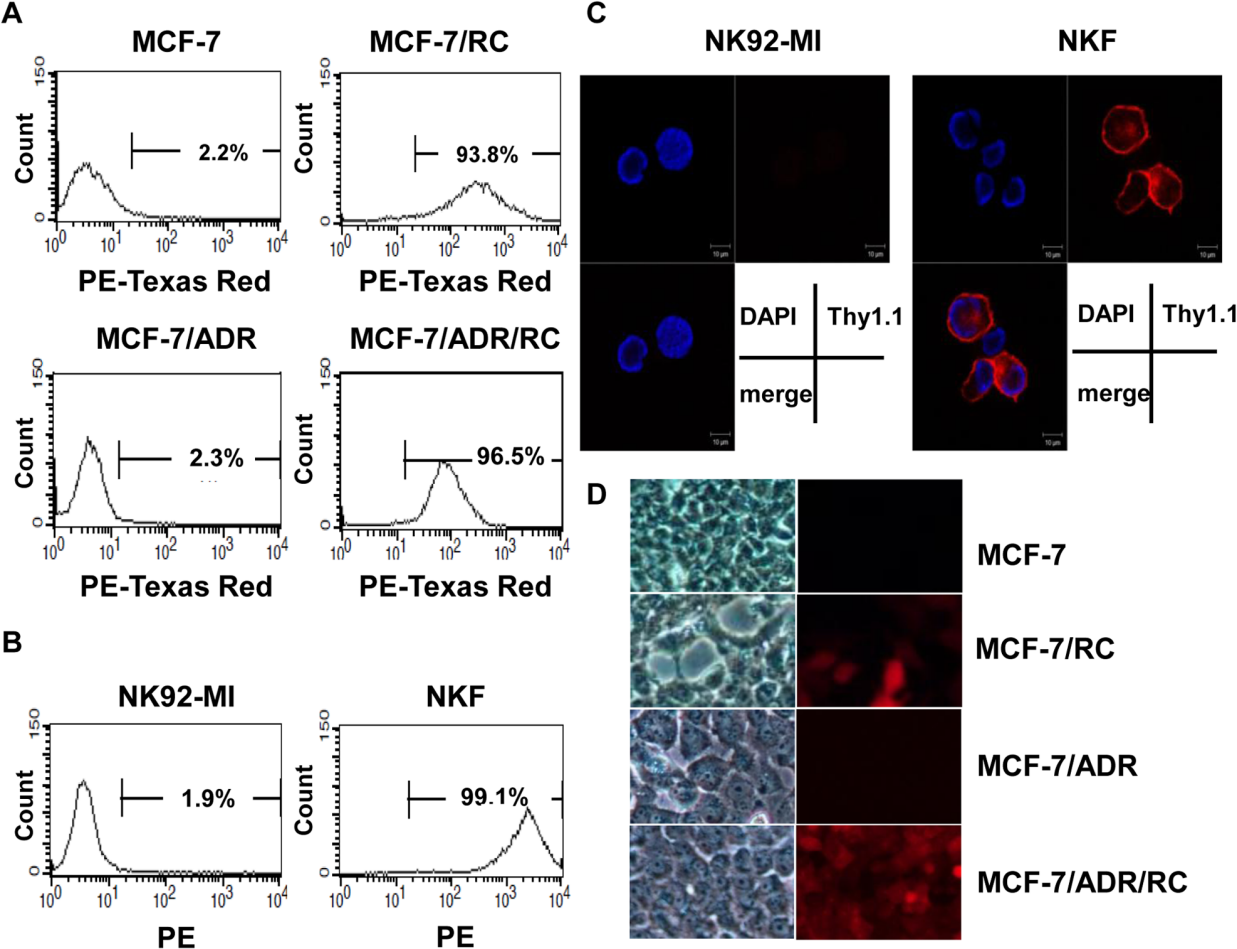
Establishment of stable cell lines expressing reporter genes. (A) Mcherry gene expression in DOX-sensitive and resistant breast cancer cells (MCF-7/RC, MCF-7/ADR/RC) was determined by using FACS.(B) effluc gene expression in NKF cell lines was determined by using FACS. PE = phycoerythrin. (C) Immunofluorescent staining of NKF cells using anti-Thy1.1-PE to assess effluc protein expression.(D) Mcherry expression under microscopy in MCF-7/RC and MCF-7/ADR/RC cells (10x).

DOX-sensitive and resistant breast cancer cells were successfully transfected with Rluc and mCherry. FACS analysis demonstrated a high expression of mCherry in MCF-7/RC and MCF-7/ADR/RC cells (Fig 1A). MCherry expression in MCF-7/RC and MCF-7/ADR/RC cells was 93.8% and 96.5%, respectively. Mcherry expression was also checked under microscopy in MCF-7/RC and MCF-7/ADR/RC cells (Fig 1D)

NK92-MI cells were transfected with a retrovirus containing the effluc gene and analyzed by flow cytometry. Following transfection, 99.1% of the cells were Thy1.1-PE positive (named NKF) and used for this experiment (Fig 1B).

Immunofluorescent staining clearly showed positive Thy1.1 expression (surrogate marker for effluc protein) in NKF cells compared to parental NK92-MI cells (Fig 1C).

### Phenotype analysis in NK and NKF cells by FACS

**Fig 2:**
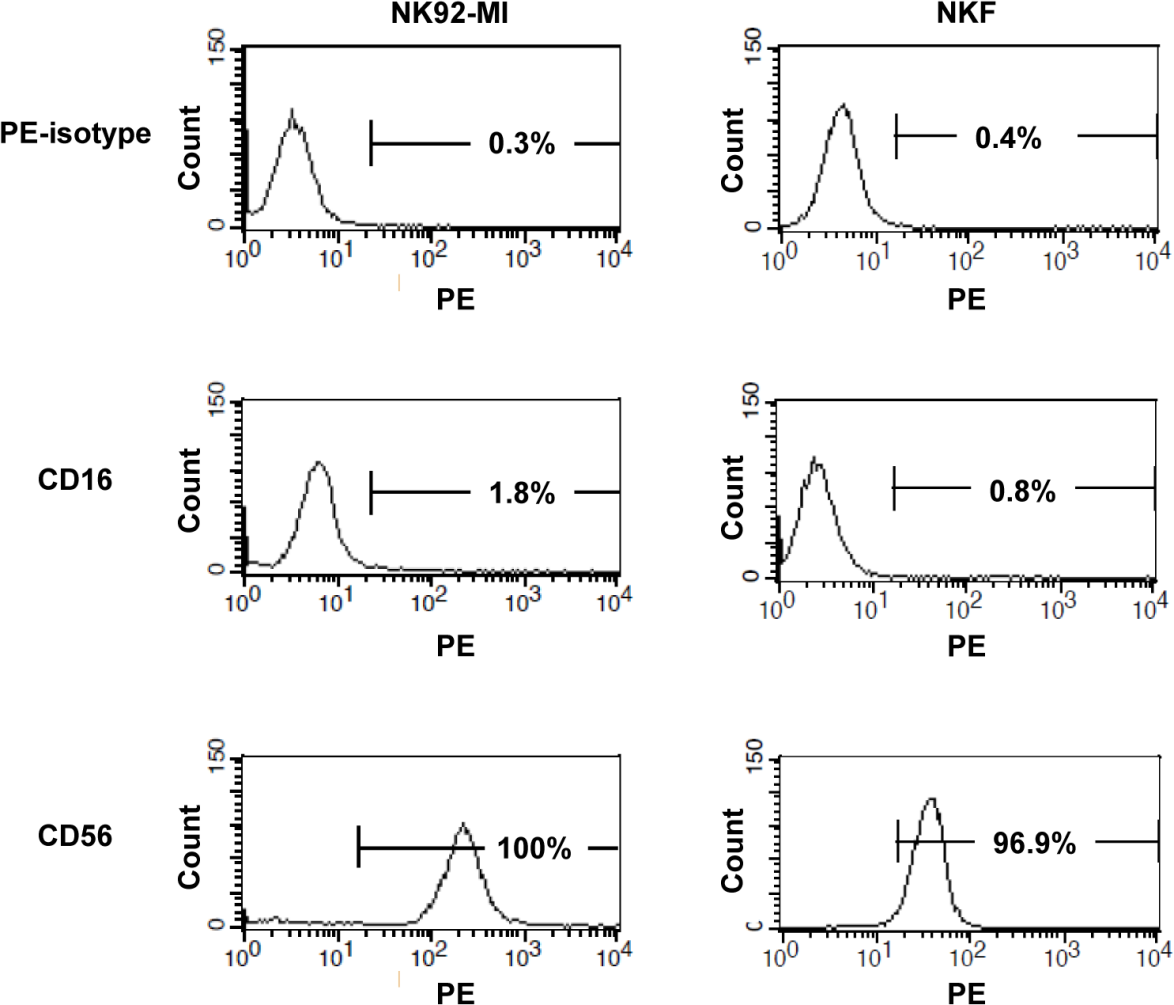
Phenotype analysis of NK92-MI and NKF cells by flow cytometry. NK92-MI and NKF cells did not express CD16. NK92-MI and NKF cells expressed CD56. Experiments were performed at least in triplicate.

Both NK and NKF cell lines expressed CD56 and were negative for CD16, which are cell surface markers. There was no significant difference between the two cell lines for the expression of these two markers (Fig 2).

### *In vivo* bioluminescence imaging

**Fig 3:**
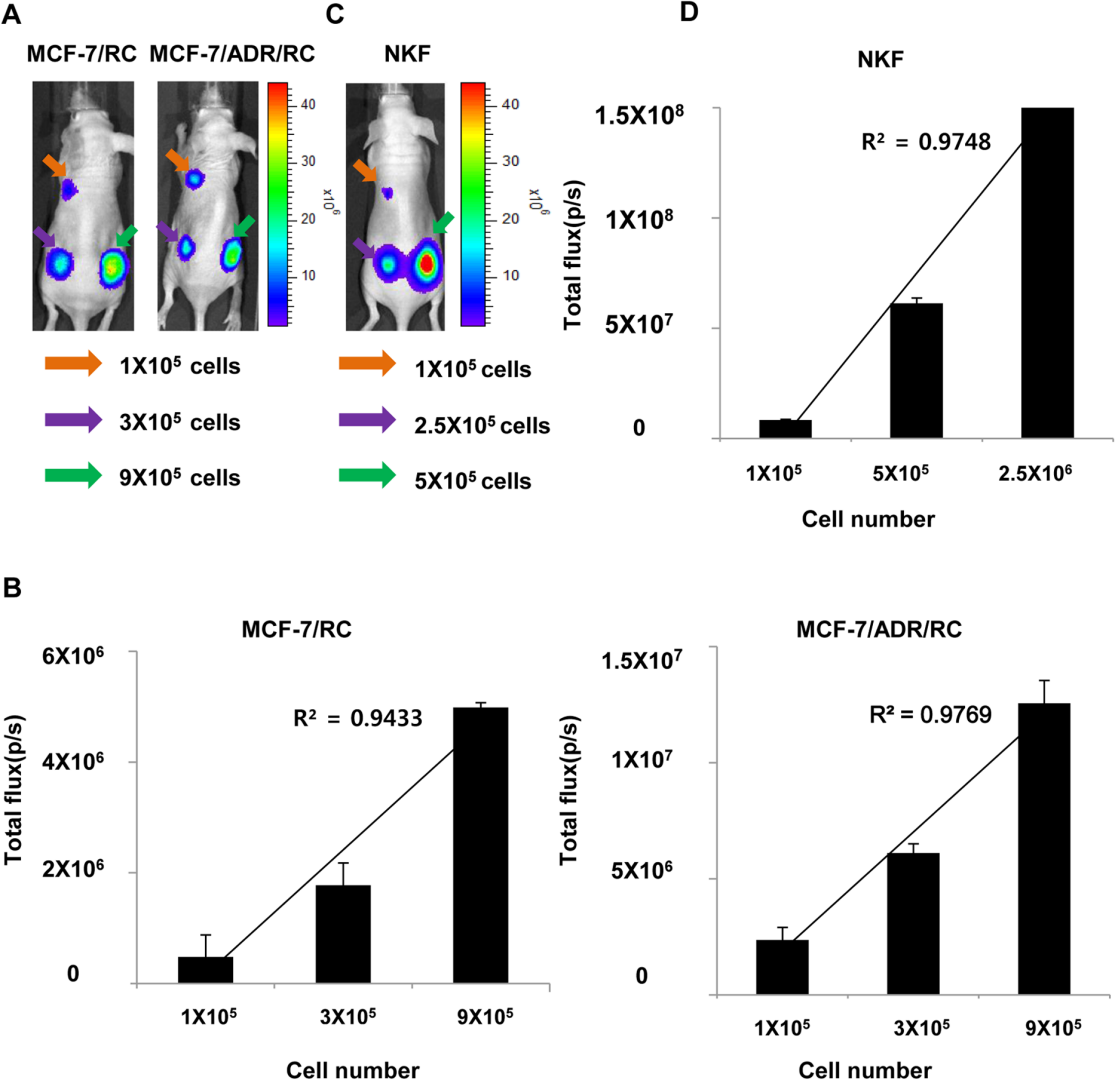
*In vivo* bioluminescence imaging. (A) Bioluminescence imaging (BLI) was performed after inoculation of different numbers of MCF-7/RC and MCF-7/ADR/RC cells into the left fore flank (Orange arrow, 1 × 10^5^ cells), left hind flank (Purple arrow, 3 × 10^5^ cells), and right hind flank (Green arrow, 9 × 10^5^ cells) of different nude mice (n = 3), respectively. (B) Average densities of BLI signals. There is a positive correlation between density and cell number of MCF-7/RC and MCF-7/ADR/RC. (C) BLI was acquired after inoculation of different number of NKF cells into the left fore flank (Orange arrow, 1 × 10^5^ cells), left hind flank (Purple arrow, 5 × 10^5^ cells), and right hind flank (Green arrow, 2.5 × 10^6^ cells) of nude mice (n = 3). (D) Average density of bioluminescence signals. There is a positive correlation between density and cell number of NKF. Experiments were performed at least in triplicate and mean values ± SD are plotted.

BLI in vivo were used to check the luciferase activity of MCF-7/RC, MCF-7/ADR/RC and NKF after establishment of reporter gene expressing stable cell lines. BLI was acquired after inoculation of MCF-7/RC and MCF-7/ADR/RC cells in the left fore-flank (1 × 10^5^), left hind-flank (3 × 10^5^), and right hind-flank (9 × 10^5^) of nude mice immediately. The signal intensities of BLI increased as the cell number increased (Fig 3A).There was a positive correlation between cell numbers and signal intensities for both cell lines (MCF-7/RC, R^2^ = 0.9433 and MCF-7/ADR/RC, R^2^ = 0.9769) (Fig 3B). *In vivo* bioluminescent images were acquired after inoculation of 1 × 10^5^, 5 ×10^5^, and 2.5 × 10^6^ NKF cells in the left fore-flank, left hind-flank, and right hind-flank of female Balbc/nude mice, respectively (Fig 3C). As shown in Fig 3D, the signal intensity from NKF cells increased with the increasing number of implanted cells and there was a positive correlation between cell numbers and signal intensities (R^2^ = 0.9748).

### NK cell cytotoxicity to MCF-7 and MCF-7/ADR cells

**Fig 4:**
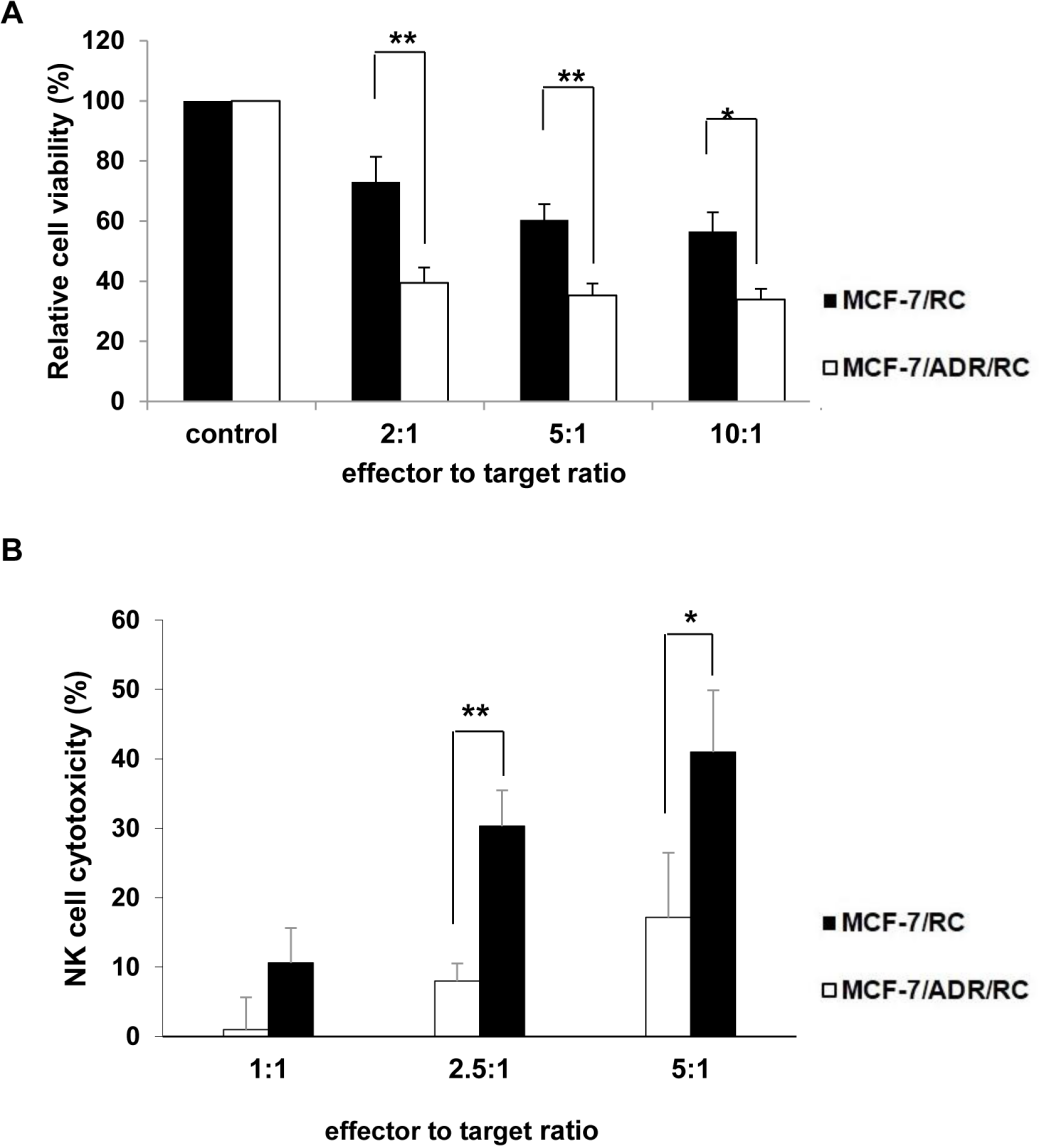
Cytolytic activity of NKF cells on MCF-7/RC and MCF-7/ADR/RC cells at different ratios. Luciferase activity of MCF-7/RC and MCF-7/ADR/RC cells decreased in an effector-number dependent manner. Both breast cancer cell lines were significantly sensitive to NKF cells. Experiments were performed at least in triplicate and mean values ± SD are plotted.

To demonstrate the therapeutic effects of NK cells *in vitro*, NKF cells were incubated with MCF-7 and MCF-7/ADR cells at different ratios. After indicate incubation, bioluminescence activity decreased as the ratio of NK cells to MCF-7/RC and MCF-7/ADR/RC increased compared to cells treated with PBS (Fig 4A). And also NK cell mediated cytotoxicity increase as the ratio of NK cells to MCF-7/RC and MCF-7/ADR/RC (Fig 4B). Additionally, the susceptibility of MCF-7/ADR cells to NK cells was higher than that of MCF-7 cells (Fig 4).

### Death receptor expression in MCF-7 and MCF-7/ADR cells

**Fig 5:**
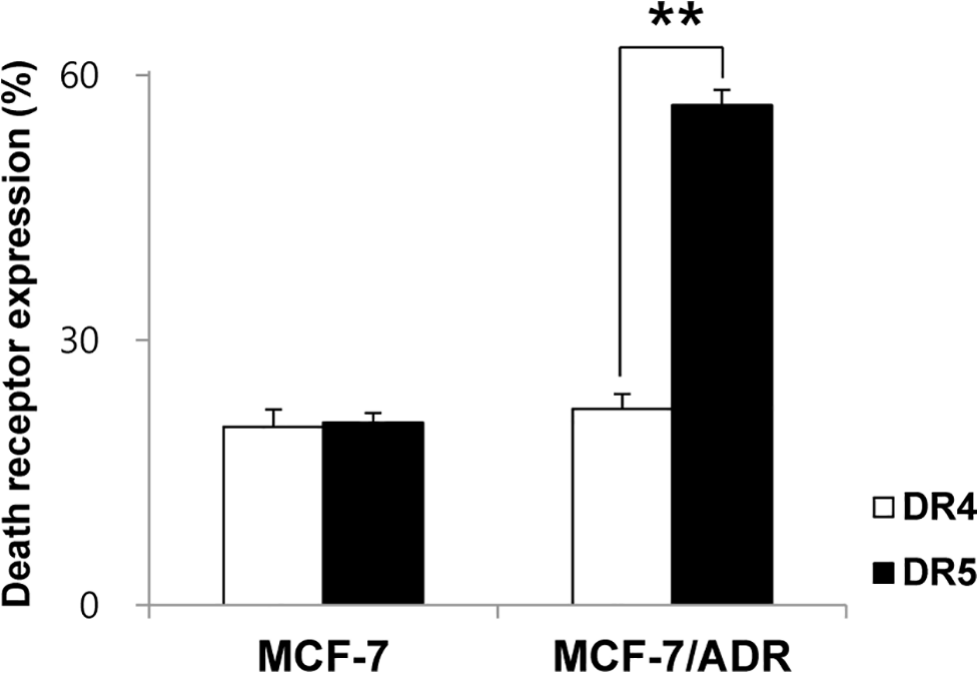
Death receptor expression at the surface of MCF-7 and MCF-7/ADR cells. Death receptor expression was determined by flow cytometry using anti-DR4-PE and anti-DR5-PE. Experiments were performed at least in triplicate and mean values ± SD are plotted.

The surface levels for DR4 and DR5 of MCF-7 and MCF-7/ADR cells were investigated by FACS analysis. DR5 level in MCF-7/ADR cell line was higher than that of the parent cell line, MCF-7 (Fig 5).

## Discussion

The present study indicates that NK cells have a therapeutic effect on DOX-resistant breast cancer cells using BLI *in vitro*.

NK92 cells are reported to have high killing effects against various cancer cells, including myeloma, leukemia, melanoma, and breast cancer, in preclinical or clinical setting [17–18]. In addition, due to its selective killing effect against cancer cells, considerable attention has been given to NK cell-based immunotherapy as a potentially effective therapeutic tool [19]. The NK-92 cell line is highly dependent on the cytokine, IL-2, and *in vivo* therapies probably require prolonged treatment with IL-2. NK-92MI, an IL-2 independent genetically modified NK-92 cell line, which has been shown to be virtually identical to the parental cell line, may be a more appropriate choice for clinical therapies because expensive exogenous IL-2 support is not required [20]. Recently, a number of clinical approaches have been used to evaluate the possibility of NK therapy in various cancers [21–22]. In the current study, we monitored the potential therapeutic effect of NK92-MI on DOX-sensitive and resistant MCF-7 breast cancer cells using a bioluminescent molecular imaging technology.

In this study, NK92-MI, MCF-7 and MCF-7/ADR cells were successfully labeled with luciferase reporter genes. BLI in vivo was performed to check whether the cells NK92-MI, MCF-7 and MCF-7/ADR stably expressing luciferase reporter gene can be monitored non-invasively in animal models for future in vivo NK cytotoxic tests. The use of bioluminescent reporter genes is an indirect cell labeling technique which is different from direct color-coded imaging with different colored fluorescent proteins and enable imaging different cell [23]. BLI was enable to accurately monitor long term survival, proliferation, and migration of labeled cells, because the reporter gene expression is maintained in daughter cells of the labeled cells, but not in nonviable cells and does not affect cell characteristics [24–25]. We found there was no significant different expression for cell surface markers of NK cell between NK92-MI and NKF cells.

In addition, the *in vivo* bioluminescent activity of both the target breast cancer cells (with Rluc) and therapeutic NK cells (with effluc) was noninvasively visualized in nude mice after subcutaneous injection using the multiplexing bioluminescent optical imaging technique. The *in vivo* multiplexing imaging strategy would be very useful in the effort to improve NK cell-based cancer therapy by visualizing both target and therapeutic cells together.

NK cells are known to kill cancer cells through various cytotoxic pathways, including death receptor ligands FasL and TRAIL. The death ligand TRAIL is expressed on NK cells and binds to the death receptors, DR4 and DR5, on tumor cells [26]. Several groups have reported that NK cell-based therapy is related to the activation of DR5-mediated apoptosis. El-Gazzar *et al*. reported that NK cell cytotoxicity was enhanced after treatment with a DR5 agonist antibody, which resulted in higher expression of DR5 in an ovarian cancer mouse model [27]. In our study, DR5 expression in MCF-7/ADR cells was higher than that of parent MCF-7 cells, which might be related with the higher sensitivity of MCF-7/ADR cells to NK cells compared to MCF-7 cells. These results support the potential effect of NK cells to overcome chemotherapy-resistance of breast cancer *in vitro*.

One of limitations of this study is the lack of *in vivo* results regarding NK cell cytotoxicity to MCF-7 and MCF-7/ADR tumors. We attempted to establish a xenograft model in nude mice using MCF-7 and MCF-7/ADR cells. Unfortunately, the production of the *in vivo* animal model was unsuccessful. However, future studies are warranted to confirm the potential of NK cell therapy for chemotherapy-resistant breast cancer.
